# Bacteria from bronchoalveolar lavage fluid from children with suspected chronic lower respiratory tract infection: results from a multi-center, cross-sectional study in Spain

**DOI:** 10.1007/s00431-017-3044-3

**Published:** 2017-12-29

**Authors:** Amparo Escribano Montaner, Juan García de Lomas, José Ramón Villa Asensi, Oscar Asensio de la Cruz, Olga de la Serna Blázquez, Mikel Santiago Burruchaga, Pedro Mondéjar López, Alba Torrent Vernetta, Yang Feng, Melissa K. Van Dyke, Janet Reyes, Pilar Garcia-Corbeira, Carla A. Talarico, on behalf of the EPI-Strep-064 study group, Silvia Castillo Corullón, Maribel Barrio Gómez de Agüero, Manuel Sánchez Solís, Antonio Moreno Galdó

**Affiliations:** 1 Pediatric Pneumology and Cystic Fibrosis Unit, University Clinic Hospital of Valencia, University of Valencia, Av. de Blasco Ibáñez, 13, 46010 Valencia, Spain; 2 Department of Microbiology, School of Medicine and University Hospital, University of Valencia, Av. de Blasco Ibañez 17, 46010 Valencia, Spain; 3 Pediatric Department, Niño Jesús University Hospital for Children, Calle Menéndez Pelayo, 65, 28009 Madrid, Spain; 4 Pediatric Pulmonology Unit, University Hospital Parc Tauli de Sabadell, Parc Taulí, 1, 08208, Sabadell, Barcelona, Spain; 5 Pediatric Department, Hospital La Paz, Paseo de la Castellana, 261, 28046 Madrid, Spain; 6 Pediatric Pneumology and Cystic Fibrosis Unit, Cruces University Hospital, Plaza de Cruces, S/N, 48903 Baracaldo, Vizcaya Spain; 7 Pediatric Pulmonology and Cystic Fibrosis Unit, Virgen of Arrixaca University Hospital, Ctra. Madrid-Cartagena, s/n, 30120, El Palmar, Murcia, Spain; 8 Paediatric Pulmonology and Cystic Fibrosis Unit, Vall d’Hebron University Hospital, Passeig de la Vall d’Hebron, 119-129, 08035 Barcelona, Spain; 9 Ningyang Group Co., Limited, C/O GSK, Wavre, Belgium; 10 GSK, Collegeville, 1250 S Collegeville Rd, Collegeville, PA 19426 USA; 11 GSK, Parque Tecnológico de Madrid, Calle de Severo Ochoa, 2, 28760 Tres Cantos, Madrid Spain; 12 GSK, Av. Fleming 20, 1300 Wavre, Belgium

**Keywords:** Chronic lower respiratory tract infection, *Streptococcus pneumoniae*, Non-typeable *Haemophilus influenzae*, Bronchoalveolar lavage, Nasopharyngeal colonization, Children

## Abstract

This cross-sectional study assessed the prevalence of bacteria isolated from Spanish children with suspected chronic lower respiratory tract infection (LRTI) for whom bronchoalveolar lavage (BAL) was indicated. BAL fluid (BALF) was collected from 191 children (aged ≥ 6 months to < 6 years, with persistent or recurrent respiratory symptoms, non-responders to usual treatment) and cultured. Nasopharyngeal swabs (NPSs) were also obtained and cultured to assess concordance of BALF and NPS findings in the same patient. *Streptococcus pneumoniae*, *Haemophilus influenzae*, or *Moraxella catarrhalis* were identified from BALF with a bacterial load indicative of infection (> 10^4^ colony-forming units/mL) in 10.5, 8.9, and 6.3% of children, respectively. Clinical characteristics were similar among participants, regardless of positivity status for any of the bacteria. Approximately 26% of pneumococcal isolates were PCV13 serotypes, and 96% of *H. influenzae* isolates were non-typeable (NTHi). Concordance between BALF and NPS isolates was 51.0% for *S. pneumoniae*, 52.1% for *H. influenzae*, and 22.0% for *M. catarrhalis*.

*Conclusion*: *S. pneumoniae*, NTHi, and *M. catarrhalis* were the main bacteria detected in BALF and NPS. Children with suspected chronic LRTI may benefit from a vaccine protecting against NTHi.

**Table:** 

**What is Known:** *• Chronic lower respiratory tract infection (LRTI) in children can cause high morbidity and is a major use of healthcare resources worldwide. Despite this, their etiology or potential preventive measures are poorly assessed.* *• Bronchoalveolar lavage can be used to determine bacterial etiology of chronic LRTI.*
**What is New:** *• We used conventional and molecular techniques to show that Haemophilus influenzae, Streptococcus pneumoniae, and Moraxella catarrhalis were present in the LRT of Spanish children with suspected chronic LRTI* *• Concordance between isolates from bronchoalveolar lavage fluid and nasopharyngeal swabs was low, suggesting that samples from the upper respiratory tract could not reliably predict the bacterial etiology of suspected chronic LRTI*

**Electronic supplementary material:**

The online version of this article (10.1007/s00431-017-3044-3) contains supplementary material, which is available to authorized users.

## Introduction

Chronic cough is associated with significant morbidity and use of healthcare resources during childhood [7, 20, 31]. A major cause of chronic cough in children is chronic bacterial lower respiratory tract infection (LRTI) and, in particular, protracted bacterial bronchitis [8, 29, 30]. Multiple infectious agents are responsible for chronic LRTI [9], but its etiology is difficult to establish, mainly because invasive techniques are required to collect specimens. When indicated, bronchoalveolar lavage (BAL) can be used for the collection of samples from the lower respiratory tract. BAL fluid (BALF) cultures have been shown to be a reliable method for determining the bacterial etiology of chronic LRTI, with non-typeable *Haemophilus influenzae* (NTHi), *Streptococcus pneumoniae*, and *Moraxella catarrhalis* being among the most commonly identified pathogens [17, 18, 30, 40, 51]. Several studies assessed a possible correlation between BALF and nasopharyngeal swab (NPS) specimens in terms of chronic LRTI etiology, with inconsistent results [26–28]. However, if evidence of a correlation between NP and BAL cultures were found, NPS could potentially be used to predict the etiology of LRTI in children with chronic respiratory symptoms.

Pediatric vaccination has significantly reduced the prevalence and carriage of these pathogens if high-coverage immunization was achieved [1, 2, 49]. *H. influenzae* type b (Hib) vaccine was introduced in the Spanish national immunization program (NIP) in 2000 [12], and vaccine coverage was ≥ 92.9% in the last decade [34]. Although routine administration of pneumococcal conjugate vaccines (PCVs) has been recommended since 2001 in Spain, not all regions originally included PCVs in their official vaccination schedule. Since 2016, PCV vaccination has been included in the infant NIP for administration at 2, 4, and 11–12 months of age [12].

In this cross-sectional study, we identified and characterized bacteria found in BALF and NPS specimens taken from Spanish children aged ≥ 6 months to < 6 years with suspected chronic LRTI and for whom a BAL procedure had been indicated. The serotype distribution of *S. pneumoniae* and *H. influenzae* isolates detected was evaluated to determine if it was covered by currently available vaccines. This study also explored correlations between findings in BALF and NPS, in order to assess whether NPS could reliably predict the etiology of chronic LRTI.

## Methods

### Study design and participants

This epidemiological, cross-sectional study was conducted between September 2013 and September 2015 in seven hospitals in Spain. Enrolled children were aged ≥ 6 months to < 6 years, had clinical suspicion of chronic LRTI, and were recommended to undergo a fiberoptic bronchoscopy with BAL. Suspected chronic LRTI was defined as persistent or recurrent respiratory signs/symptoms not responding to usual treatment (receipt of bronchodilators and inhaled corticosteroids for at least 3 months, repeated cycles of antibiotics up to 15 days in duration): wet cough lasting > 4 weeks without additional associated symptoms, recurrent (≥ 3 episodes per year) or persistent (lasting > 3 months) wheezing, persistent pathologic auscultation (lasting > 4 weeks), or persistent (> 1 month) or recurrent (≥ 3 occurrences per year) infiltrates/atelectasis observed on chest radiograph. Exclusion criteria from enrolment were as follows: child in care, exacerbation of persistent respiratory symptoms or receipt of antibiotic treatment within 2 weeks prior to enrolment, known cystic fibrosis, immunosuppression, or other severe immunodeficiencies.

BALF was collected while children were under sedation with topical anesthesia, according to routine procedure [13], choosing the most most-affected lobe (as identified radiologically or by fiberoptic bronchoscopy) or the right middle lobe as the standard sampling lobe. The volume of lavage solution was calculated according to international guidance [13] and three samples were obtained for each BAL. NPS was taken at the same time the BAL was performed. Data on demographics and clinical characteristics were collected from interviews with parents or legally acceptable representatives (LARs), children’s medical records, and routine physical examinations performed during the visit.

The study was approved by Independent Ethics Committees from each hospital, and a written informed consent was obtained from the children’s parents or LARs. The study is registered at Clinicaltrials.gov (NCT02838407).

### Study objectives

The primary objective was to identify and characterize the bacterial etiology in BALF specimens taken from children with suspected chronic LRTI, visiting the hospital for a BAL procedure. As previously described [26, 27, 30, 51], a bacterial load of *S. pneumoniae*, *H. influenzae*, or *M. catarrhalis* > 10^4^ colony-forming units (CFU)/mL was considered indicative of infection when only one organism was identified in BALF; this cut-off was > 10^5^ CFU/mL if more than one organism was present.

Secondary objectives included assessing the presence of bacterial pathogens with any bacterial load as detected by culture from BALF and NPS; characterizing *S. pneumoniae*, *H. influenzae*, and *M. catarrhalis* isolates identified in the upper and lower respiratory tract and assessing possible correlations between them; and describing the study population in terms of demographics and clinical characteristics.

### Microbiology

The second aliquot of each wash was used for culture. Samples of ≥ 2 mL of BALF were stored in skim-milk tryptone glucose glycerol transport/storage medium [22]. NPS specimens were stored in the same medium [44]. All specimens were maintained at ≤− 70 °C until tested.

Pure and 10^−3^ to 10^−5^ dilutions of BALF and NPS specimens were seeded and duplicated in three bacteriological media: blood agar-gentamicin (5 mg/L) [45], chocolate agar-bacitracin (5000 U/L) [35] (incubated at 37 °C in 5% CO_2_), and MacConkey agar (incubated at 37 °C without CO_2_). Plates were observed at 24 and 48 h, and colony counting was performed to calculate the CFU/mL. Standard bacteriological methods (including Gram staining, catalase, oxidase, X and V factor requirement, optochin, bile solubility or DNAse test, depending of the phenotypic, and Gram stain characteristics of the colonies) were used to identify *H. influenzae*, *S. pneumoniae*, *M. catarrhalis*, and any other presumptive bacterial pathogens.

In addition, bacterial identification was confirmed by qualitative real-time polymerase chain reaction (RT-PCR) and bacterial load was determined by quantitative RT-PCR using *lgtC*, *lytA*, and *copB* genes for *H. influenzae*, *S. pneumoniae*, and *M. catarrhalis*, respectively [3, 6, 24, 25].

A duplex RT-PCR using *lgtC* and *P6* genes as targets was performed to confirm the identification of NTHi and to differentiate from *H. haemolyticus*. *S. pneumoniae* serotyping was performed by passive agglutination test (Pneumotest latex kit) and confirmed by Quellung reaction using specific factor antisera (Statens Serum Institute, Copenhagen) or by PCR [37]. *H. influenzae* types were determined using polyvalent, a-, b-, c-, d-, e-, and f-specific antisera.

Susceptibility testing of *H. influenzae*, *S. pneumoniae*, and *M. catarrhalis* against penicillin, erythromycin azithromycin, tetracycline, levofloxacin, trimethoprim/sulfamethoxazole, and amoxicillin/clavulanate was performed by broth microdilution following the procedures recommended by The Clinical and Laboratory Standards Institute (CLSI) [11]. A β-lactamase test was performed to all *H. influenzae* and *M. catarrhalis* isolates using the commercial Nitrocephin [36] and the acidimetric [48] tests. To detect *H. influenzae* β-lactamase-non-producing ampicillin-resistant isolates, ampicillin resistance was tested by broth microdilution for all β-lactamase negative isolates. The classification of isolates as susceptible, intermediately susceptible, and resistant was based on minimum inhibitory concentrations according to the CLSI [11].

Two isolates of *H. influenzae*, *S. pneumoniae*, or *M. catarrhalis* selected per specimen type per child were compared to determine whether they were clones of each other or different (unique) organisms. Isolates were defined to be unique based on the comparison of serotype and antimicrobial susceptibility for *S. pneumoniae* and serotypable *H. influenzae*; uniqueness was defined based on the antibiogram alone for NTHi and *M. catarrhalis*. If the two isolates per specimen type per child had different serotypes, or the same serotype but different susceptibility patterns, they were considered unique and both were retained in the analyses.

### Statistical analyses

Analyses were conducted on all evaluable children with available BALF or NPS specimens. Proportions of *S. pneumoniae*, *H. influenzae*, *M. catarrhalis*, and other bacteria detected by culture in BALF specimens and in NPS were computed with associated exact 95% confidence intervals (CIs). Serotype/type and/or antibiotic susceptibility distributions (number and percentage) were determined for isolates of *S. pneumoniae*, *H. influenzae*, and *M. catarrhalis* detected in BALF cultures meeting the cut-off for infection, and the same analyses were performed *post hoc* for BALF cultures with any bacterial load and NPS specimens. Concordance between BALF and NPS specimens for *H. influenzae*, *S. pneumoniae*, and *M. catarrhalis* was assessed descriptively as the number and percentage of unique isolates with positive results in both specimens for each bacterium*.*

Analyses were descriptive and were performed using the Statistical Analysis Systems v9.2.

## Results

### Study participants

A total of 197 children were enrolled, of whom six were eliminated from the analyses (two children not meeting the age criteria for inclusion, and four with receipt of antibiotic treatment within 2 weeks prior to enrollment). The mean age of participants was 39.8 months, and 48.2% were girls. In total, 88.0% of children had received ≥ 1 dose of any PCV and 72.3% had received ≥ 3 doses of PCV13; 95.3% of children had received ≥ 3 vaccine doses against Hib; and 80.1% had received antibiotics in an interval of time of < 6 months and > 2 weeks prior to enrollment (Table 1).

**Table 1:** Demographic and clinical characteristics of the study participants

	Evaluable participants (*N* = 191)
Age (mean ± SD), months	39.84 ± 17.76
Female, *n* (%)	92 (48.2)
Weight (mean ± SD), kg	14.67 ± 3.83
Height (mean ± SD), cm	96.04 ± 13.10
Age distribution, *n* (%)	
6–11 months	14 (7.3)
12–23 months	29 (15.2)
24–35 months	32 (16.8)
36–47 months	38 (19.9)
48–59 months	52 (27.2)
60–71 months	26 (13.6)
Indications observed, *n* (%)	
Cough	151 (79.1)
Wheezing	86 (45.0)
Pathologic auscultation	74 (38.7)
Infiltrates/atelectasis (diagnosed by X-ray)	131 (68.6)
Any pre-existing respiratory conditions, *n* (%)	
Asthma	72 (37.7)
Pneumonia	114 (59.7)
Bronchiolitis	85 (44.5)
Bronchiectasis	15 (7.9)
Bronchitis	143 (74.9)
Rhinitis	15 (7.9)
Pre-existing otitis media conditions, *n* (%)	
Acute otitis media	53 (27.8)
Otitis media with effusion or “glue ear”	6 (3.1)
Otitis media with perforation and discharge	8 (4.2)
Relevant vaccination history, *n* (%)	
Any PCV^a^	168 (88.0)
≥ 2 doses of PCV7	13 (6.8)
≥ 2 doses of PCV13 or PHiD-CV	120 (62.8)
Hib vaccine, ≥ 2 doses	186 (97.4)
Influenza vaccine, ≥ 1 dose	58 (30.4)
Antibiotic taken^b^, *n* (%)	153 (80.1)
Penicillin	83 (43.5)
Amoxicillin/ clavulanate	76 (39.8)
Azithromycin	57 (29.8)
Cephalosporins	24 (12.6)
Clarithromycin	8 (4.2)
Other^c^	6 (3.0)

*N*, number of children included in the analyses; SD, standard deviation; *n* (%), number (percentage) of children in each category; PCV, pneumococcal conjugate vaccine; PCV7/13, 7/13-valent PCV; PHiD-CV, pneumococcal polysaccharide non-typeable *Haemophilus influenzae* protein D-conjugate vaccine; Hib, *Haemophilus influenzae* type b

^a^Two children received one dose of a polysaccharide pneumococcal vaccine and four doses of PCV13 at subsequent vaccinations

^b^In the period ≥ 2 weeks and < 6 months prior to enrollment in the study

^c^Other antibiotics were ciprofloxacin (taken by two children) and erythromycin, clindamycin, linezolid, and meropenem (each taken by one child)

### Culture and bacterial load

#### BALF

*S. pneumoniae*, *H. influenzae*, and *M. catarrhalis* were detected in cultures with any bacterial load in 30.5, 51.1, and 49.5% of children, respectively, and ≥ 1 of the three bacteria was detected by microbiological cultures in 74.2% of BALF specimens. *S. pneumoniae*, *H. influenzae*, and *M. catarrhalis* met the cut-off for infection in BALF specimens from 10.5, 8.9, and 6.3% of children, respectively (Table 2). These percentages were in line with those obtained by using by quantitative RT-PCR as method of detection. Co-infections with two and three of the three bacteria were detected in seven and zero children, respectively. Other bacterial organisms detected by culture growth were *Staphylococcus aureus* (in one child), *Pseudomonas aeruginosa* (in five children), *Stenotrophomonas maltophilia* (in four children), and *Pseudomona putida* (in two children).

**Table 2:** Bacterial etiology in BALF and NPS specimens

	Cultures with any bacterial load, % (95% CI)		Cultures meeting the cut-off for infection^a^, % (95% CI)
	BALF (*N* = 190)	NPS (*N* = 191)	BALF (*N* = 190)
*S. pneumoniae*	30.5 (24.1–37.6)	51.8 (44.5–59.1)	10.5 (6.5–15.8)
*H. influenzae*	51.1 (43.7–58.4)	46.6 (39.4–53.9)	8.9 (5.3–13.9)
*M. catarrhalis*	49.5 (42.2–56.8)	56.0 (48.7–63.2)	6.3 (3.3–10.8)
Any of the three	74.2 (67.4–80.3)	80.6 (74.3–86.0)	22.1 (16.4–28.7)
Other pathogens	6.3	3.7	–

%, percentage of children in each category; CI, confidence interval; *N*, number of children with available results; BALF, bronchoalveolar lavage fluid; NPS, nasopharyngeal swab

^a^Bacterial load > 10^4^ CFU/mL if the pathogen was present alone or > 10^5^ CFU/mL if present as co-infection

#### NPS

*S. pneumoniae*, *H. influenzae*, and *M. catarrhalis* were detected in cultures from 51.8, 46.6, and 56.0% of children, respectively (Table 2). Other bacterial pathogens identified were *S. aureus* (in six children) and *S. maltophilia* (in one child).

### Clinical characteristics

Similar frequencies of cough, wheezing, pathologic auscultation, and infiltrates/atelectasis were observed in children with or without BALF cultures indicative of infection with *S. pneumoniae*, *H. influenzae*, or *M. catarrhalis*; a similar observation was made for fibro-bronchoscopy indications and respiratory conditions (Table 3). The clinical characteristics and medical records for the children in whom the other bacteria were detected were similar to that of the entire study population.

**Table 3:** Summary of clinical characteristics of children with BALF cultures meeting the cut-off for infection, by organism positivity status

	*S. pneumoniae*		*H. influenzae*		*M. catarrhalis*	
	Positive	Negative	Positive	Negative	Positive	Negative
	*N* = 20	*N* = 170	*N* = 17	*N* = 173	*N* = 12	*N* = 178
Indication observed in the patient, *n* (%)						
Cough	14 (70.0)	136 (80.0)	16 (94.1)	134 (77.5)	10 (83.3)	140 (78.7)
Wheezing	5 (25.0)	80 (47.1)	6 (35.3)	79 (45.7)	6 (50.0)	79 (44.4)
Pathologic auscultation	9 (45.0)	64 (37.7)	6 (35.3)	67 (38.7)	6 (50.0)	67 (37.6)
Infiltrates/atelectasis (diagnosed by X-ray)	12 (60.0)	119 (70.0)	12 (70.6)	119 (68.8)	7 (58.3)	124 (69.7)
Main fibro-bronchoscopy indication in patients, *n* (%)						
Wet cough for > 4 weeks without associated symptoms^a^	5 (25.0)	57 (33.5)	4 (23.5)	58 (33.5)	2 (16.7)	60 (33.7)
Persistent wheezing (lasting > 3 months)^a^	1 (5.0)	7 (4.1)	0 (0.0)	8 (4.6)	0 (0.0)	8 (4.5)
Recurrent wheezing (≥ 3 episodes/year)^a^	2 (10.0)	23 (13.5)	2 (11.8)	23 (13.3)	3 (25.0)	22 (12.4)
Persistent pathologic auscultation (lasting > 4 weeks)^a^	3 (15.0)	13 (7.7)	2 (11.8)	14 (8.1)	2 (16.7)	14 (7.9)
Persistent infiltrates/atelectasis (lasting > 1 month)^a^	8 (40.0)	57 (33.5)	8 (47.1)	57 (33.0)	4 (33.3)	61 (34.3)
Recurrent infiltrates/atelectasis (≥ 3 occurrences/year)^a^	1 (5.0)	13 (7.7)	1 (5.9)	13 (7.5)	1 (8.3)	13 (7.3)
Pre-existing respiratory conditions, *n* (%)						
Pneumonia	13 (65.0)	101 (59.4)	10 (58.8)	104 (60.1)	8 (66.7)	106 (59.6)
Bronchiolitis	8 (40.0)	77 (45.2)	11 (64.7)	74 (42.8)	4 (33.3)	81 (45.5)
Bronchiectasis	2 (10.0)	13 (7.7)	1 (5.9)	14 (8.1)	0 (0.0)	15 (8.4)
Bronchitis	16 (80.0)	126 (74.1)	12 (70.6)	130 (75.1)	9 (75.0)	133 (74.7)
Rhinitis	1 (5.0)	14 (8.2)	0 (0.0)	15 (8.7)	1 (8.3)	14 (7.9)

BALF, bronchoalveolar lavage fluid; *N*, number of children with available results; *n* (%), number (percentage) of children in each category

^a^Not responding to usual treatment

Blood sample results were available for 109 (57.1%) children, for whom the mean white blood cell count was 540.2 × 10^9^/L; the mean C-reactive protein level, 25.5 mg/L; and the mean erythrocyte sedimentation rate, 25.3 mm/h. A chest X-ray was done for 166 (86.9%) children; an abnormal result (images of persistent/recurrent atelectasis-consolidation in the same or different lobules) was detected in 143 (74.9%) of them, and for 122 (85.3%), the abnormality was considered by the investigator as relevant to the decision to collect BALF.

### Serotype distribution

**Fig. 1:**
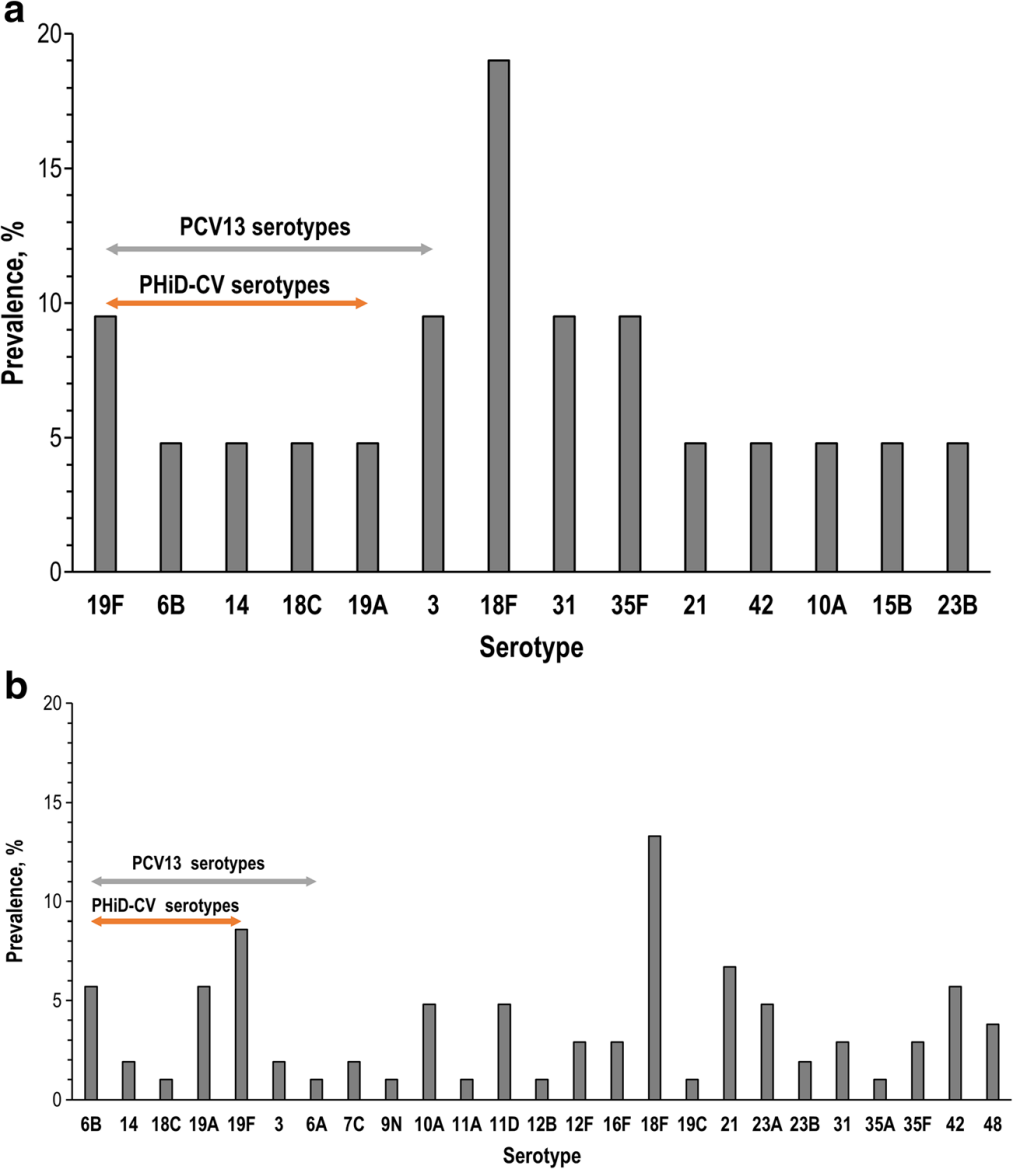
Serogroup and serotype distribution of *S. pneumoniae*-positive children for cultures meeting the cut-off for infection from BALF (**a**; *N* = 21) and cultures of any load from NPS (**b**; *N* = 105); BALF, bronchoalveolar lavage fluid; NPS, nasopharyngeal swab; *N*, number of unique isolates from children with positive quantitative identification for *S. pneumoniae*; PHiD-CV, pneumococcal polysaccharide non-typeable *Haemophilus influenzae* protein D-conjugate vaccine; PCV13, 13-valent pneumococcal conjugate vaccines

Among 65 unique isolates from BALF cultures with any bacterial load of *S. pneumoniae*, 10 (15.4%) were positive for serotype 18F, 17 (26.2%) for ≥ 1 PCV13 serotype (Fig. 1a, b and S1), and four (6.2%) for non-typeable *S. pneumoniae*. Among 21 *S. pneumoniae* isolates from BALF cultures meeting the cut-off for infection, 14 pneumococcal serotypes were identified, with serotype 18F having the highest prevalence (in four (19.0%) isolates). Six PCV13 vaccine serotypes (3, 6B, 14, 18C, 19A, and 19F) were present in 38.1% of these cultures (Fig. 1a).

Of 98 unique isolates from BALF cultures with any bacterial load of *H. influenzae*, 95 (96.9%) were positive for NTHi, and three (3.0%) for type f. Among the 18 unique *H. influenzae* isolates from cultures meeting the cut-off for infection, NTHi was identified in 17 (94.4%), while type f was detected in one (5.6%).

In NPS specimens, ≥ 1 PCV13 serotype was detected in 25.7% of isolates, and serotype 18F had the highest prevalence (13.3%) (Fig. 1b), while among the 92 isolates of *H. influenzae*, 95.7% were positive for NTHi and 4.3% for type f.

### Antimicrobial susceptibility

Antimicrobial susceptibility of isolates from BALF and NPS specimens is presented in Table 4.

**Table 4:** Antimicrobial profile of unique isolates identified in BAL and NPS specimens

Antibiotic	Cultures with any bacterial load						Cultures meeting the cut-off for infection^a^		
	BALF			NPS			BALF		
	*S*	*I*	*R*	*S*	*I*	*R*	*S*	*I*	*R*
*S. pneumoniae*	*N* = 65			*N* = 105			*N* = 21		
Penicillin, *n* (%)	36 (55.4%)	13 (20.0%)	16 (24.6%)	55 (52.4%)	27 (25.7%)	23 (21.9%)	15 (71.4%)	2 (9.5%)	4 (19.0%)
Amoxicillin/clavulanate, *n* (%)	50 (76.9%)	7 (10.8%)	8 (12.3%)	86 (81.9%)	10 (9.5%)	9 (8.6%)	17 (81.0%)	3 (14.3%)	1 (4.8%)
Erythromycin, *n* (%)	43 (66.2%)	1 (1.5%)	21 (32.3%)	55 (52.4%)	1 (1.0%)	49 (46.7%)	17 (81.0%)	0 (0.0%)	4 (19.0%)
Azithromycin, *n* (%)	41 (63.1%)	3 (4.6%)	21 (32.3%)	57 (54.3%)	4 (3.8%)	44 (41.9%)	15 (71.4%)	2 (9.5%)	4 (19.0%)
Tetracycline, *n* (%)	51 (78.5%)	14 (21.5%)	0 (0.0%)	71 (67.6%)	3 (2.9%)	31 (29.5%)	18 (85.7%)	0 (0.0%)	3 (14.3%)
Levofloxacin, *n* (%)	65 (100%)	0 (0.0%)	0 (0.0%)	103 (98.1%)	0 (0.0%)	2 (1.9%)	21 (100%)	0 (0.0%)	0 (0.0%)
TMP/SMX, *n* (%)	24 (36.9%)	13 (20.0%)	28 (43.1%)	52 (49.5%)	13 (12.4%)	40 (38.1%)	3 (14.3%)	5 (23.8%)	13 (61.9%)
MDR, *n* (%)	33 (50.8%)			59 (56.2%)			7 (33.3%)		
*H. influenzae*	*N* = 98			*N* = 92			*N* = 18		
Penicillin, med (min, max)	0.5 (0.13, 16.00)			0.5 (0.13, 16.00)			0.50 (0.25, 16.00)		
Amoxicillin/clavulanate, *n* (%)	91 (92.9%)	0 (0.0%)	7 (7.1%)	91 (98.9%)	0 (0.0%)	1 (1.1%)	15 (83.3%)	0 (0.0%)	3 (16.7%)
Erythromycin, med (min, max)	4 (1.00, 16.00)			4.00 (0.25, 16.00)			4.00 (2.00, 16.00)		
Azithromycin, *n* (%)	95 (96.9%)	0 (0.0%)	3 (3.1%)	89 (96.7%)	0 (0.0%)	3 (3.3%)	17 (94.4%)	0 (0.0%)	1 (5.6%)
Tetracycline, *n* (%)	98 (100%)	0 (0.0%)	0 (0.0%)	92 (100%)	0 (0.0%)	0 (0.0%)	18 (100%)	0 (0.0%)	0 (0.0%)
Levofloxacin, *n* (%)	98 (100%)	0 (0.0%)	0 (0.0%)	92 (100%)	0 (0.0%)	0 (0.0%)	18 (100%)	0 (0.0%)	0 (0.0%)
TMP/SMX, *n* (%)	51 (52.0%)	16 (16.3%)	31 (31.6%)	49 (53.3%)	18 (19.6%)	25 (27.2%)	5 (27.8%)	7 (38.9%)	6 (33.3%)
Beta-lactamase positive, *n* %	7 (7.1%)			9 (9.8%)			3 (16.7%)		
MDR, *n* (%)	7 (7.1%)			6 (6.5%)			4 (22.2%)		
*M. catarrhalis*	*N* = 98			*N* = 110			*N* = 13		
Penicillin, med (min, max)	8.00 (0.02, 16.00)			16.00 (0.02, 16.00)			16.00 (0.50, 16.00)		
Amoxicillin/clavulanate, *n* (%)	95 (96.9%)	0 (0.0%)	3 (3.1%)	105 (95.5%)	0 (0.0%)	5 (4.5%)	12 (92.3%)	0 (0.0%)	1 (7.7%)
Erythromycin, *n* (%)	90 (91.8%)	0 (0.0%)	8 (8.2%)	106 (96.4%)	0 (0.0%)	4 (3.6%)	12 (92.3%)	0 (0.0%)	1 (7.7%)
Azithromycin, *n* (%)	84 (85.7%)	0 (0.0%)	14 (14.3%)	106 (96.4%)	0 (0.0%)	4 (3.6%)	11 (84.6%)	0 (0.0%)	2 (15.4%)
Tetracycline, *n* (%)	98 (100%)	–	0 (0.0%)	110 (100%)	0 (0.0%)	0 (0.0%)	13 (100%)	0 (0.0%)	0 (0.0%)
Levofloxacin, *n* (%)	98 (100%)	–	0 (0.0%)	110 (100%)	0 (0.0%)	0 (0.0%)	13 (100%)	0 (0.0%)	0 (0.0%)
TMP/SMX, *n* (%)	56 (57.1%)	35 (35.7%)	7 (7.1%)	103 (93.6%)	0 (0.0%)	7 (6.4%)	7 (53.8%)	5 (38.5%)	1 (7.7%)
Beta-lactamase positive, *n* (%)	93 (94.9%)			108 (98.2%)			13 (100%)		
MDR, *n* (%)	10 (10.2%)			5 (4.5%)			2 (15.4%)		

BALF, bronchoalveolar lavage fluid; NPS, nasopharyngeal swab; *S*, susceptible; *I*, intermediate; *R*, resistant; *N*, number of unique isolates for each pathogen; *n* (%), number (percentage) of children in each category; TMP/SMX, trimethoprim/sulfamethoxazole; MDR, multi-drug resistance; med, median; min, minimum; max, maximum

^a^Bacterial load > 10^4^ CFU/mL if the pathogen was present alone or > 10^5^ CFU/mL if present as co-infection

### Concordance between BAL and NP isolates

Concordance between isolates from BALF and NPS specimens from children with positive bacterial cultures was established for 25 (51.0%) of 49 pneumococcal isolates, 37 (52.1%) of 71 *H. influenzae* isolates, and 18 (22.0%) of 82 *M. catarrhalis* isolates. Concordance between specimens meeting the cut-off for infection was lower (Tables 5 and 6).

**Table 5:** Concordance between microbiological culture growth results from BALF and NPS specimens in participants with any bacterial load and with bacterial loads meeting the cut-off for infection

	Isolates with any bacterial load, *n* (%)			Isolates with a bacterial load meeting the cut-off for infection^a^, *n* (%)		
	*S. pneumoniae N* = 49	*H. influenzae* *N* = 71	*M. catarrhalis* *N* = 82	*S. pneumoniae* *N* = 30	*H. influenzae* *N* = 15	*M. catarrhalis* *N* = 18
BALF	49 (100%)	71 (100%)	82 (100%)	20 (66.7%)	12 (80.0%)	11 (61.1%)
NPS	49 (100%)	71 (100%)	82 (100%)	18 (60.0%)	6 (40.0%)	10 (55.6%)
Both BALF and NPS	49 (100%)	71 (100%)	82 (100%)	8 (26.7%)	3 (20.0%)	3 (16.7%)
Concordance	25 (51.0%)	37 (52.1%)	18 (22%)	2 (6.7%)	2 (13.3%)	1 (5.6%)

*N*, number of unique isolates; *n* (%), number (percentage) of unique isolates present in each specimen type; BALF, bronchoalveolar lavage fluid; NPS, nasopharyngeal swab; CI, confidence interval; PPV, positive predictive value; NPV, negative predictive value

^a^Bacterial load > 10^4^ CFU/mL if the pathogen was present alone or > 10^5^ CFU/mL if present as co-infection

**Table 6:** Measures of agreement between microbiological culture growth results from BALF and NPS specimens with any bacterial load and with bacterial loads meeting the cut-off for infection

	Isolates with any bacterial load			Isolates with a bacterial load meeting the cut-off for infection^a^		
	*S. pneumoniae*	*H. influenzae*	*M. catarrhalis*	*S. pneumoniae*	*H. influenzae*	*M. catarrhalis*
BALF, *n*	58	97	94	20	17	12
NPS, *n*	98	89	106	98	89	106
Both BALF and NPS, *n*	49	71	83	20	12	11
Sensitivity, value (95% CI)	0.84 (0.73–0.93)	0.73 (0.63–0.82)	0.88 (0.80–0.94)	1.00 (0.83–1.00)	0.71 (0.44–0.90)	0.92 (0.62–1.00)
Specificity, value (95% CI)	0.63 (0.54–0.71)	0.81 (0.71–0.88)	0.76 (0.66–0.84)	0.54 (0.46–0.62)	0.55 (0.48–0.63)	0.47 (0.39–0.54)
PPV, value (95% CI)	0.50 (0.40–0.60)	0.80 (0.70–0.88)	0.78 (0.69–0.86)	0.20 (0.13–0.30)	0.13 (0.07–0.22)	0.10 (0.05–0.18)
NPV, value (95% CI)	0.90 (0.82–0.95)	0.74 (0.65–0.82)	0.87 (0.78–0.93)	1.00 (0.96–1.00)	0.95 (0.89–0.98)	0.99 (0.94–1.00)

BALF, bronchoalveolar lavage fluid; NPS, nasopharyngeal swab; *n*, number of isolates with positive growth/or meeting the cut-off for infection; CI, confidence interval; PPV, positive predictive value; NPV, negative predictive value

^a^Bacterial load > 10^4^ CFU/mL if the pathogen was present alone or > 10^5^ CFU/mL if present as co-infection

## Discussion

In this study in children aged ≥ 6 months to < 6 years with suspected chronic LRTI, *S. pneumoniae* was detected in 10.5% of BALF cultures with bacterial loads indicative of infection, while a prevalence of 8.9% for *H. influenzae* and 6.3% for *M. catarrhalis* was estimated. The observed prevalence of BALF findings indicative of infection and co-infection was lower than that reported in the literature.

There is a lack of consensus on a cut-off to differentiate a lower airway infection from upper respiratory tract contamination when culturing BALFs. Whereas 10^5^ CFU/mL is the usual cut-off for pediatric cystic fibrosis patients [4], several studies propose values of 10^3^ (in adult patients) or 10^4^ CFU/mL [51] for pediatric chronic LRTI. Similar definitions for a load indicative of infection to the one used in our study was previously used [26, 27, 30, 51]. In a study in 30 non-indigenous young children with persistent respiratory symptoms in Australia using the same cut-off for infection, *S. pneumoniae* was identified as the major pathogen in the BALF of 23% of participants, followed by *M. catarrhalis* in 17% and NTHi in 7% of participants [26]. In another study in 197 children from the USA with persistent wet cough using ≥ 10^4^ CFU/mL as a threshold for infection, NTHi was isolated in BALF cultures in 49% of children, while *S. pneumoniae* and *M. catarrhalis* were identified in 20 and 17% of children [51], in agreement with prior findings using the ≥ 10^5^ CFU/mL cut-off [30]. Similar observations were made in 104 Indigenous Australian children with bronchiectasis, of whom 31% had NTHi infections, compared to 16% for *S. pneumoniae* and 12% for *M. catarrhalis* [27]. Bacterial co-infections were observed in our study in < 4% of BALF specimens, while prevalences of up to 35.4% have been reported [30, 40]. Our study did not identify any correlation between clinical characteristics of patients and presence of bacterial cultures with a load > 10^4^ CFU/mL, likely due to the small sample size. However, our results confirmed previous findings establishing *S. pneumoniae*, NTHi, and *M. catarrhalis* as the main organisms in the BALF of children with suspected chronic LRTI. Of note, most children had received antibiotic treatment within 6 months prior to sampling, and this could have impacted the BALF results, especially if the antibiotic was taken within the interval of 2–4 weeks prior to procedure. This could explain the lower rates of infection and co-infection observed in our study compared to previous studies.

Although most children in our study received ≥ 2 doses of PCV13, serotypes 6B, 14, 18C, 19A, and 19F (common to both PCV13 and the pneumococcal polysaccharide protein D-conjugate vaccine PHiD-CV) and serotype 3 (PCV13 serotype) were present in 38.9% of BALF cultures with bacterial load indicative of infection and 26.2% of BALF cultures with any bacterial load. In NPS specimens, PCV13 serotypes were detected in 25.7% of isolates (serotypes 3, 6A, 6B, 19A, and 19F). Currently, two PCVs are used in pediatric immunization programs worldwide: a 10-valent PCV with cross-reactive protection to serotype 19A (PHiD-CV; [47]) and a 13-valent PCV (PCV13; [39]). Although PCV13 is recommended in the Spanish NIP, PHiD-CV is also authorized for use in children < 5 years of age. PHiD-CV has been shown to have a similar overall impact on invasive pneumococcal disease as PCV13 [15, 46], and the protein D from NTHi included as carrier component in PHiD-CV may provide additional protection against this organism [10]. Recent studies have reported a prevalence of up to 57% of PCV13 serotypes causing invasive disease from 2010 to 2014, in regions of Spain where vaccine coverage was estimated at around 55% [16, 33], together with an emergence of serotype 12F [16]. The most prevalent pneumococcal serotype identified in our study in both BALF and NPS specimens from children with suspected chronic LRTI was 18F, which is not included in either PCV. Serotype 18F is not frequently reported as a cause of invasive pneumococcal or mucosal disease, even in the post-PCV era [33]. The high prevalence of this serotype in our study may be due to local epidemiology/outbreak of a certain 18F strain, and genetic analyses are warranted to confirm this hypothesis.

In our study, *H. influenzae* was identified with bacterial loads indicative of infection in 8.9% of BALF specimens. The epidemiology of *H. influenzae* has changed following the widespread use of Hib vaccination during infancy, and NTHi has become more prevalent in both carriage and disease [50]. The introduction of PHiD-CV in routine childhood immunization schedule could help to protect children against NTHi carriage (and subsequent aspiration into lower respiratory tracts), since previous studies have shown a reduction of NTHi nasopharyngeal colonization in vaccine recipients compared with control groups [14, 41]. Moreover, PHiD-CV vaccination was associated with an improved immune response to NTHi in children with chronic suppurative lung disease [38].

A lack of concordance in findings from BALF and NPS specimens was evident in our study; the concordance was even poorer for specimens meeting the cut-off for infection, although the threshold of > 10^4^ CFU/mL might not be relevant for NP carriage. These results seem to support previous observations that NP sampling of the upper respiratory tract does not necessarily correlate well with bacterial pathogens causing lower airway infections [4, 26, 42] and suggesting that NPS cannot replace BALF testing for children with suspected chronic LRTI. It is also possible that a combined assessment of NP and oropharyngeal microbiota of the upper respiratory tract would be better predictors of lower respiratory tract bacterial flora [32], bearing in mind that false positives due to contamination are possible with this type of specimens.

Most bacterial isolates detected in this study were susceptible to the tested antibiotics. This result was not surprising, as the high resistance rates previously reported in Spain [23] have fallen, and at present, *S. pneumoniae*, *H. influenzae*, and *M. catarrhalis* have low levels of resistance, similar to other European countries [19, 21, 43]. This observation is likely due to judicious use of antibiotics with respect to dosage, duration of treatment, and improved targeting of prescriptions for the correct indication.

Our study has several limitations. Most children received antibiotics within the 6 months prior to enrolment, which could have resulted in the low prevalence of bacteria from BALF meeting the diagnostic cut-off for infection and also potentially resulted in a lack of concordance between the findings from BALFs and NPs (e.g., if antibiotic treatment differently affected the BALF and NP flora). The BALF was collected from a single lung lobe, and neither BAL cytology nor measurement of inflammatory mediators was performed for these samples. In addition, the samples were not screened for viral pathogens which might have caused clinical presentations similar to those which constituted inclusion criteria for enrolment in our study; therefore, bacteria could not be definitely established as the only cause of the suspected LRTI. Although PCR assays can increase the sensitivity of organism detection vs. conventional culture techniques, to date, there is no PCR method which can unequivocally differentiate NTHi and *H. haemolyticus* [5], and this could constitute another potential limitation of our study.

## Conclusions

*S. pneumoniae*, NTHi, and *M. catarrhalis* were identified as the main bacterial organisms in both BALF and NPS specimens from children with suspected chronic LRTI. Clinical characteristics were similar among study participants, regardless of the presence of any of the three pathogens in bacterial loads indicative of infections in BALF. Despite most participants being vaccinated with at least three PCV13 doses, PCV13 serotypes were recovered in approximately 25% of *S. pneumoniae*-positive BALF and NPS specimens.

A lack of concordance in findings in BALF and NPS from the same patient was observed, indicating that NPS cannot be used to predict the bacterial etiology in children with suspected chronic LRTI.

Future vaccination policies should take into account the etiology of chronic LRTI in children, and our results suggest that vaccination against NTHi disease may contribute to the prevention of LRTI.

## Electronic supplementary material

ESM 1 BALF, bronchoalveolar lavage fluid; N, number of unique isolates from children with positive identification for S. pneumoniae (any load); PHiD-CV, pneumococcal polysaccharide non-typeable Haemophilus influenzae protein Dconjugate vaccine; PCV13, 13-valent pneumococcal conjugate vaccines (JPEG 89.6 kb)

High resolution image (TIFF 185 kb)

## Abbreviations

BAL: Bronchoalveolar lavage
BALF: Bronchoalveolar lavage fluid
CFU: Colony-forming units
CI: Confidence interval
CLSI: The Clinical and Laboratory Standards Institute
LAR: Legally acceptable representative
LRTI: Lower respiratory tract infection
NIP: National immunization program
NPS: Nasopharyngeal swab
NTHi: Non-typeable *Haemophilus influenzae*
PCV: Pneumococcal conjugate vaccine
RT-PCR: Real-time polymerase chain reaction
