# Supplementary figures and images for: Bacteria from bronchoalveolar lavage fluid from children with suspected chronic lower respiratory tract infection: results from a multi-center, cross-sectional study in Spain
Source: Eur J Pediatr. 2017 Dec 29;177(2):181–92. doi: 10.1007/s00431-017-3044-3 (PMC5758651; doi:10.1007/s00431-017-3044-3)

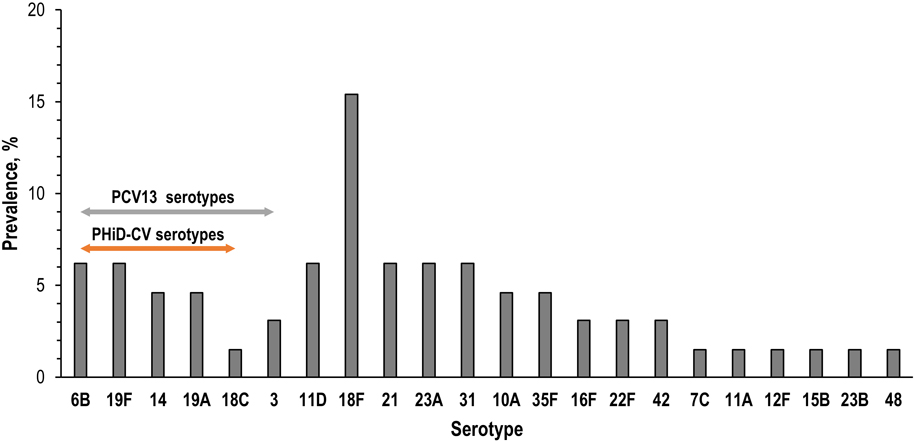

Supplement: Supplementary file 1 — BALF, bronchoalveolar lavage fluid; N, number of unique isolates from children with positive identification for S. pneumoniae (any load); PHiD-CV, pneumococcal polysaccharide non-typeable Haemophilus influenzae protein Dconjugate vaccine; PCV13, 13-valent pneumococcal conjugate vaccines (JPEG 89.6 kb) [file 431_2017_3044_Fig2_ESM.jpg]

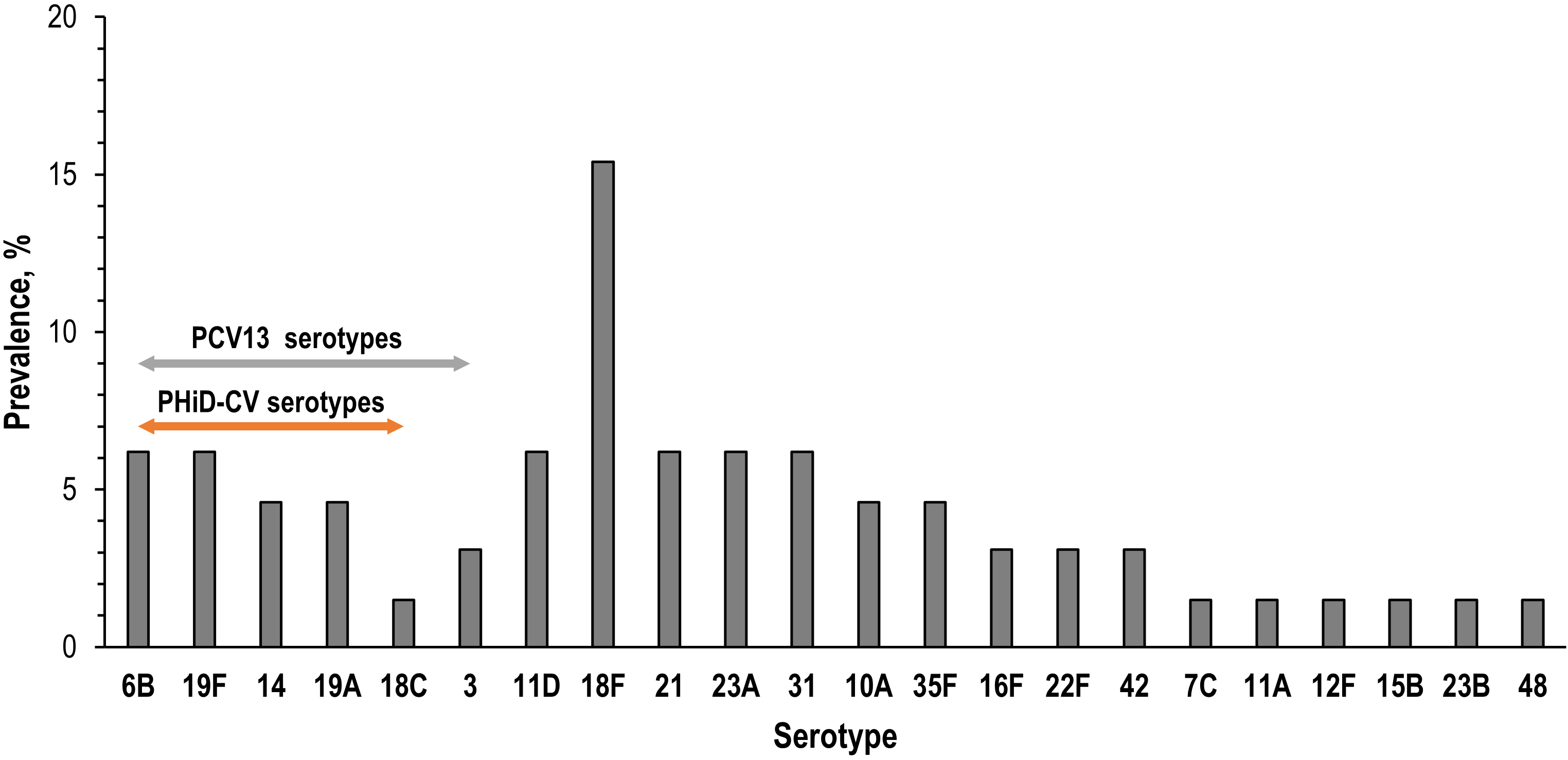

Supplement: Supplementary file 2 — High resolution image (TIFF 185 kb) [file 431_2017_3044_MOESM1_ESM.tiff]
